# Transcriptomic response to parasite infection in Nile tilapia (*Oreochromis niloticus*) depends on rearing density

**DOI:** 10.1186/s12864-018-5098-7

**Published:** 2018-10-01

**Authors:** Amy R Ellison, Tamsyn M Uren Webster, Olivier Rey, Carlos Garcia de Leaniz, Sofia Consuegra, Pablo Orozco-terWengel, Jo Cable

**Affiliations:** 1 School of Biosciences, Cardiff University, Cardiff, CF10 3AX UK; 2 Biosciences Department, Swansea University, Swansea, SA2 8PP UK; 3 Present address: Université Perpignan Via Domitia, IHPE UMR 5244, CNRS, IFREMER, University Montpellier, F-66860 Perpignan, France

**Keywords:** Stocking density, Stress, *Saprolegnia*, Aquaculture, Transcriptome, Tilapia, Circadian rhythm, Disease risk

## Abstract

**Background:**

Captive animal populations, be it for food production or conservation programmes, are often maintained at densities far beyond those in natural environments, which can have profound effects on behaviour, immune and stress levels, and ultimately welfare. How such alterations impact transcriptional responses to pathogen infection is a ‘different kettle of fish’ and remains poorly understood. Here, we assessed survival and gene expression profiles of infected fish reared at two different densities to elucidate potential functional genomic mechanisms for density-related differences in disease susceptibility.

**Results:**

Utilising a whole-transcriptome sequencing (RNAseq) approach, we demonstrate that rearing density in tilapia (*Oreochromis niloticus*) significantly impacts susceptibility to the oomycete *Saprolegnia parasitica*, via altered transcriptional infection responses. Tilapia held at low densities have increased expression of genes related to stress, likely due to increased aggressive interactions. When challenged with *Saprolegnia*, low-density fish exhibit altered expression of inflammatory gene responses and enhanced levels of adaptive immune gene suppression compared to fish reared at higher density, resulting in significantly increased mortality rates. In addition, *Saprolegnia* infection substantially perturbs expression of circadian clock genes, with fish reared at low-density having higher levels of molecular clock dysregulation.

**Conclusions:**

Our results reveal the wide-scale impact of stocking density on transcriptional responses to infection and highlight the need to incorporate circadian biology into our understanding of disease dynamics in managed animals.

**Electronic supplementary material:**

The online version of this article (10.1186/s12864-018-5098-7) contains supplementary material, which is available to authorized users.

## Background

Increasing global human populations are escalating spatial conflicts for food production. As competition for land and water grows, food security and sustainable intensification of food production remain at the forefront of worldwide political agendas [1]. In the past, rearing densities in intensive food animal production were primarily optimised for maximal growth and/or productivity, and hence economic benefit [2]. Today, however, there is increasing consideration of animal rearing density on behaviour [3, 4], stress levels [5, 6], immune levels [5, 7], and ultimately welfare [3, 6].

Intensification of aquaculture increasingly relies on maintaining fish stocks at densities vastly different to those found in natural populations. Stocking/rearing density and disease management are considered pivotal factors determining fish farm productivity and profitability [8–10]. In fish, sub-optimal rearing density can increase cortisol levels, likely due to altered social interactions [11, 12]. Wide-scale transcriptional differences due to stocking conditions (potentially due to chronic social stress) appear to include a number of immunologically important pathways [7]. However, critically, it is unknown how these transcriptome-level alterations translate to actual infection responses and thus disease susceptibility. Examining the effects of rearing density on disease susceptibility in fish at a transcriptomic level will not only provide critical insight for effective disease management in aquaculture, but also for other managed animal populations such as terrestrial livestock and captive breeding programmes for conservation.

Infections due to *Saprolegnia* oomycetes result in significant economic losses in fish farming [13]*. S. parasitica*, the species most commonly associated with fish saprolegniasis, typically elicits strong host inflammatory responses, yet is capable of actively suppressing several components of the adaptive immune system including T-helper cell cytokines, antigen presentation machinery and immunoglobulins [14]. For tilapia aquaculture, second only to carp in global economic importance [15], devastating outbreaks typically occur in outdoor rearing facilities during cooler months, resulting in high mortality and severe economic losses [16, 17]. Although malachite green is highly effective at controlling saprolegniasis, since its worldwide ban in 2002 there are limited effective treatment options, leading to a global resurgence of the disease [18]. It is therefore essential that conditions influencing susceptibility to *Saprolegnia* infection are elucidated to effectively manage risks and reduce reliance on pharmaceutical interventions in aquaculture.

Extreme high fish densities increase plasma cortisol in tilapia, which is typically taken as evidence for chronic stress [19]. However, in some fish species, including tilapia, increased levels of aggression between individuals are observed at lower densities [11, 20] which may also act as a potent chronic social stressor. We hypothesise that a sub-optimal, low rearing density in tilapia will significantly impact the expression of immune gene pathways, leading to a decreased resistance to infection challenge. In this study, we experimentally challenged juvenile Nile tilapia (*Oreochromis niloticus*), reared under two densities, to *S. parasitica* and compared infection resistance and skin and gills transcriptome-wide gene expression (quantified via RNAseq) between uninfected and infected fish. We use our data to characterise the wider functional genomic effects of stocking density on disease responses and make recommendations for the improvement of approaches used in the management of disease dynamics in captive animals.

## Results

### Stocking density and disease-challenge survival

**Fig. 1:**
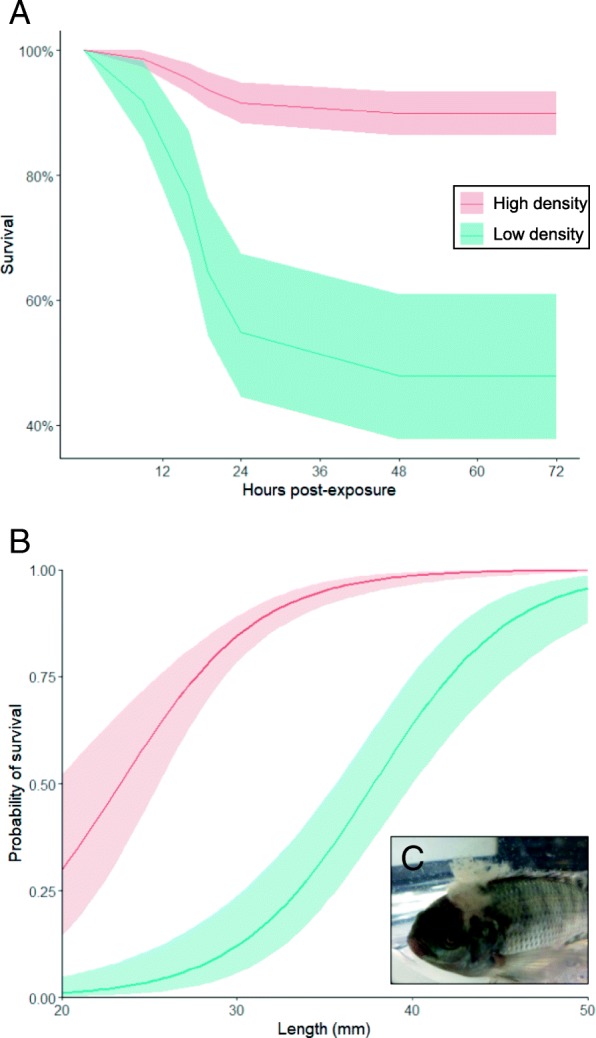
**a** Cox’s proportional hazards survival model of *Oreochromis niloticus* challenged with *Saprolegnia parasitica* at two stocking densities. Shading indicates 95% CI bands. **b** Logistic regression predicting probability of survival during *Saprolegnia* infection by fish standard length. Shading indicates 95% CI bands. **c** Example of *Saprolegnia* infection on *Oreochromis niloticus*

Susceptibility to *Saprolegnia* infection (i.e. mortality due to infection) was examined in tilapia raised under two density conditions (high-density = 6 kg m^− 3^, low-density = 1.5 kg m^− 3^). No mortality was observed in sham-challenged (uninfected/control) fish in either high- (*n* = 80) or low-density (*n* = 20) treatments for the duration of the experiment. Overall mortality was 10.2 and 52.1% in high- (*n* = 29/285) and low-density (*n* = 38/73) *Saprolegnia*-challenged treatments respectively. Stepwise survival model selection resulted in a final model including density, length and their interaction, excluding body condition and its interactions with other variables (Additional file 1). In the final model, host survival following *Saprolegnia* challenge was significantly lower in fish maintained at low density (Cox Hazard Ratio HR = 28.631, *P* < 0.001; Fig. 1a). In addition, smaller fish were significantly less likely to survive infection (HR = 23.355, *P* < 0.001) and there was a significant interaction between density treatment and fish length (HR = 0.151, *P* = 0.026; Fig. 1b). Maintaining fish at low density significantly increased average fish length (Welch’s *t* = − 2.28, *P* = 0.023).

### Differential gene expression in uninfected fish

To examine the transcriptomic effects of stocking density in uninfected fish, we compared gene expression between sham-challenged (uninfected/control) fish maintained at high and low density at two time points (24 and 48 h post-exposure). In the gill, 2672 and 1268 genes were differentially expressed between uninfected high- and low-density fish at 24 and 48 h respectively. Genes with significantly higher expression levels in low-density fish at both timepoints (*n* = 154) were enriched for biological processes related to vitamin transport (e.g. GO:0051180: vitamin transport, GO:0032218: riboflavin transport) and included folate transporter 1 (*folt*) and riboflavin transporter 2 (*rft2*). Biosynthesis/metabolism genes (e.g. GO:0044210: de novo CTP biosynthetic process, GO:0006541: glutamine metabolic process) were also more highly expressed, including CTP synthase 1 (*ctps1*) and amidophosphoribosyltransferase (*ppat*). The most significantly enriched gene ontology (GO) terms of genes more highly expressed at low density were found to be related to circadian processes (e.g. GO:0032922: circadian regulation of gene expression, GO:0009648: photoperiodism), including period (*per3*) and casein kinase I isoform delta (*csnk1d*). In addition, upregulated genes in low-density fish gills included “steroid hormone signalling” (GO:0003707, e.g. *nr1d2, paqr7*) and “response to stress” (GO:0006950, e.g. *hspa9, hspd1, trap1*). Genes with higher expression in the gills of fish maintained at high density (*n* = 165) were enriched for biological processes related to lipid and nitrogen compound transport (e.g. GO:0015917: aminophospholipid transport, GO:0010876: lipid localisation), including phospholipid-transporting ATPase (*atp8a2*) and oxysterol binding like protein 2 (*osbpl2*). A complete list of enriched GO terms from gill tissues is provided in Additional file 2.

In the skin, 6171 and 2810 genes were differentially expressed between uninfected high- and low-density fish at 24 h and 48 h respectively. Genes exhibiting higher expression levels in low-density fish at both timepoints (*n* = 474) were highly enriched for processes related to metabolism and biosynthesis (e.g. GO:0006629: lipid metabolic process, GO:0016053: organic acid biosynthetic process), including several lipid synthases (*fasn, isyna1, pdss1, ptgs1*). Similar to the gills, genes with higher expression in low-density fish also included several GO terms related to circadian processes (GO:0009648: photoperiodism, GO:0007623: circadian rhythm; e.g. *per3*). Genes with higher expression in the skin of fish maintained at high density (*n* = 644) were enriched for biological processes related to T cells (e.g. GO:0045580 regulation of T cell differentiation; *gata3*), skin structure (e.g. GO:0098773 skin epidermis development; *nrg1*) and protein ubiquitination (GO:0016567; *rnf19a*). A complete list of enriched GO terms from skin tissues is provided in Additional file 3.

**Fig. 2:**
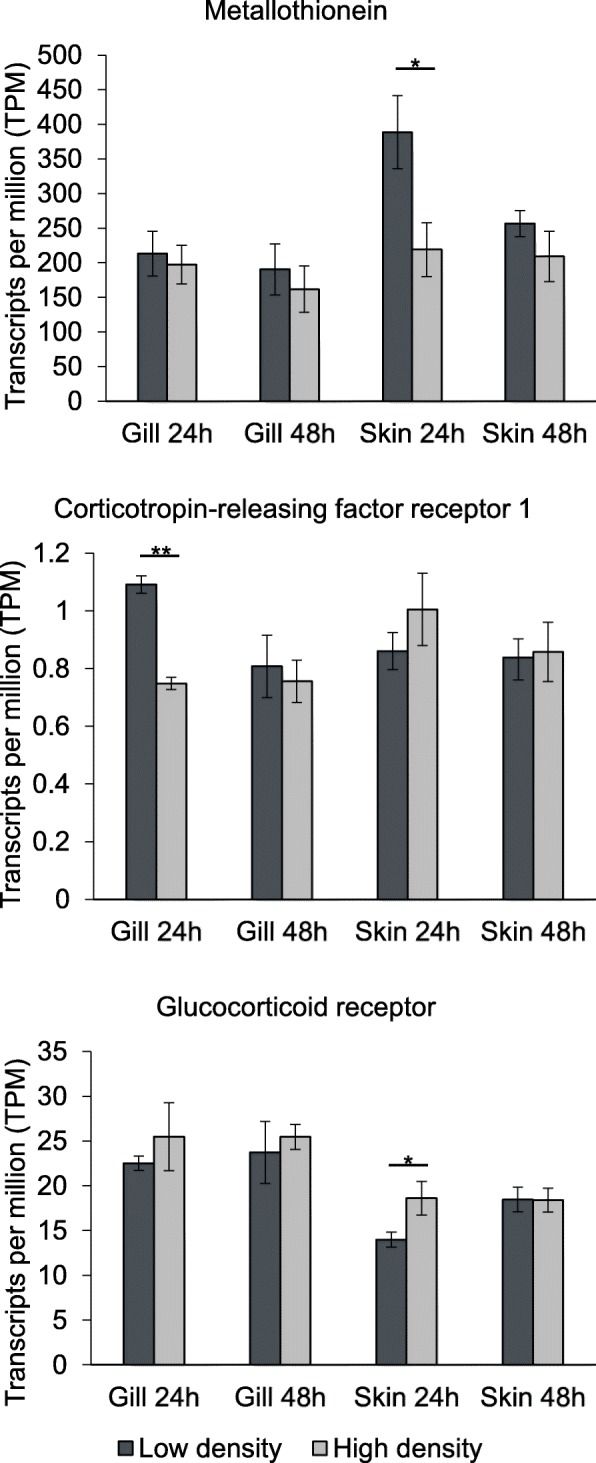
Differential expression of stress-related genes in uninfected *Oreochromis niloticus* at two stocking densities. Asterisks denote significant differences in expression (contrasts between tissues and/or time points not shown)

Genes previously identified as markers of stress and/or cortisol production in fish and other vertebrates were specifically compared between uninfected fish at high and low density in both skin and gill tissues. In the gills, corticotropin-releasing factor receptor 1 (*crhr1*) was more highly expressed in low density fish at 24 h. In the skin of low density fish, metallothionein (*mt*) showed higher expression and glucocorticoid receptor (*nr3c1*) reduced expression at 24 h (Fig. 2). Furthermore, two skin gene co-expression modules (SK4, SK13; Additional file 4) significantly associated with density (higher expression in low-density fish) included significant enrichment of GO terms related to stress responses such as “cellular response to stress” (SK4; e.g. *dmap1*) and “cortisol secretion” (SK13; e.g. *ptpn11*).

### Gene expression responses to Saprolegnia infection shared between densities

The numbers of genes found to be differentially expressed among control (uninfected) and *Saprolegnia*-challenged fish and their GO term enrichments are summarised in Additional files 5 and 6. In gill tissues, a total of 175 genes were more highly expressed in high and low density infected fish at both time points (24 and 48 h post-exposure). This gene set was highly enriched for metabolic processes including glucose (GO:0006006) and lipid (GO:0006629) metabolism (e.g. *hk2, eno1*). Genes downregulated in all infected fish groups were predominantly enriched by GO terms related to circadian processes (Additional file 6), but also included “MHC class II protein binding” (GO:0042289; e.g. *cd74*). Shared early responses (24 h) to *Saprolegnia* included increased gene expression related to osmotic stress (GO:0006970, e.g. *slc31a1*), epidermal repair (GO:0048730, e.g. *tp63*), mucous secretion (GO:0070254, e.g. *p2ry2*), and response to fungus (GO:0009620, e.g. *ncf1*). Eight gene co-expression modules were significantly associated with infection, but not density (Additional file 8). Three of the four co-expression modules (G1, G4, G5) negatively correlated with infection status (i.e. lower expression in infected fish) and were enriched for immune response terms including “antigen processing and presentation” (GO:0019882, e.g. *cd83*, MHC II alpha and beta chains) and “interleukin-12 production” (GO:0032615, e.g. *mast2*) (Additional file 8). Two of the four modules positively correlated with infection status were enriched for a number of innate and adaptive immune response terms (Additional file 8), including both T cell and mast cell processes (e.g. *rac1, rc3h1*).

In the skin, genes with decreased expression in infected individuals at both densities were highly enriched for immune functions. The most significant GO term for the 75 genes found to exhibit lower expression in infected fish in both density treatments and time points was “immune response” (GO:0006955) and were also enriched for “interferon-gamma-mediated signalling pathway” (GO:0060333) Additional file 10. This gene set included suppressor of cytokine signalling (*socs1*), beta-2-microglobulin (*b2m*), T-cell surface glycoprotein CD4 (*cd4*), and C-C motif chemokine 17 (*ccl17*). In addition, genes sharing decreased expression in infected fish in both density treatments, during at least one time point, were enriched for a number of adaptive immune terms including “MHC class II protein complex” (GO:0042613, e.g. HLA class 2 gamma chain, H-2 class 2 alpha chain) and “T cell activation” (GO:0042110, e.g. *pag1, gata3*) (Additional file 5). The 148 genes found to have higher expression in the skin of infected fish from both time points and density treatments were not significantly enriched for immune related functions. However, shared early responses to infection (24 h post-exposure in both densities) included increased expression of genes associated with inflammation and wound healing (e.g. mast cell migration, epithelial cell migration, interleukin-1 receptor activity; Additional file 5). In the weighted gene co-expression network analysis (WGCNA), no association between co-expression gene modules and density treatment was found, although overall nine gene modules were significantly associated with infection (Additional file 7). Modules positively correlated with infection status (i.e. increased expression in infected individuals) included enrichment for mast cell mediated immune pathways (SK10, SK11, SK14).

### Density-specific responses to Saprolegnia infection

**Fig. 3:**
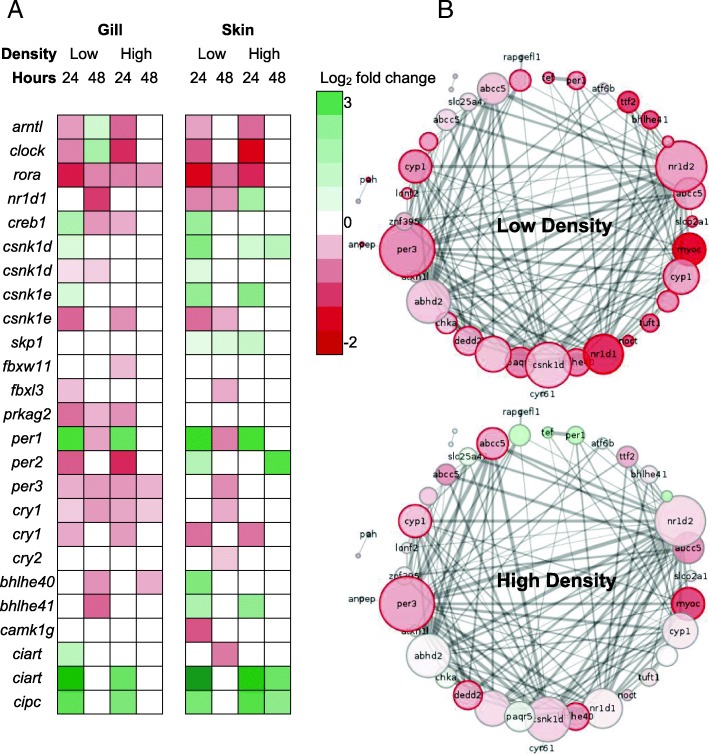
**a** Differential expression of circadian rhythm genes between uninfected and *Saprolegnia*-infected *Oreochromis niloticus*. Positive (green) and negative (red) log_2_ fold changes indicate higher and lower expression in infected fish respectively. Zero change (white) indicates no significant difference in expression. **b** Network view of G9 gill module (circadian regulation of gene expression) hub genes. Nodes are labelled with official gene symbols when available. Edge line width represents connection strength (weight); thicker lines denote stronger connections. Node size is proportional to number connections. Node colour represents the log2 fold change between control and infected fish at 48 h post-exposure. Node borders represent differential gene expression results. Grey = nonsignificant; red = decreased expression in the infected; green = increased expression in the infected

To examine the potential transcriptomic underpinnings for the differences in disease susceptibility between stocking densities, we identified differentially expressed genes unique to each density treatment. In the gills, enrichment of inflammatory response regulation was found in genes with significantly increased expression in low-density infected fish at 24 h (e.g. *tnip1, sbno2*, Additional file 6). In contrast, increased gene expression at 24 h unique to high-density fish was enriched for a number of GO terms related to immunoglobulin-mediated immune responses (e.g. *msh2, batf*, Additional file 6). Furthermore, we found enrichment of GO terms related to T cell functions in genes with increased expression only in infected fish at high-density at both timepoints (e.g. *trpm4*, Additional files 6 and 13). Downregulated gill genes in low-density fish, at both timepoints, were enriched for terms related to major histocompatibility complex (MHC) II (e.g. MHC class II beta chain), complement and humoral immune responses (e.g. *masp1*, Additional file 6). Only a single gene co-expression module was significantly correlated with both infection status (lower expression in infected fish) and density treatment (lower expression in low-density fish) in the gills (G9; Additional files 8 and 11). This module of 127 genes (including *per3*, *csnk1d*, *ciart*) was predominantly enriched for GO terms related to circadian functions (e.g. circadian regulation of gene expression), indicating significant disruption of molecular clock expression due to infection (Fig. 3).

In the skin, similar to early responses of the gill, responses to infection unique to low density fish at 24 h (*n* = 1965) were enriched for increased expression of inflammatory pathways (e.g. *il17c,* Additional files 5 and 9). In contrast, early responses (24 h) of infected high-density fish (*n* = 905) were enriched for metallopeptidase activity (e.g. *mmp28*, Additional file 5). In addition, while suppression of immune pathways, particularly adaptive immune responses, were found in the skin at both densities, only the low-density group were found to have decreased collagen biosynthetic activity (e.g. *p4ha1*, Additional file 5). Gene co-expression modules SK1 and SK6, enriched for adaptive immune responses (SK1; e.g. *klhl6, trpm4*) and wound healing processes (SK6; e.g. *cask*, Additional file 7), were negatively correlated with infection status and positively associated with density, indicating a lower level of immunosuppression in the skin of high density fish.

## Discussion

Here we demonstrate that sub-optimal stocking levels significantly alter fish disease responses at the transcriptional level, and result in higher disease susceptibility. Specifically, we found that tilapia raised at low densities are likely under chronic social stress [11, 20] (evidenced by increased expression of markers related to cortisol production and other stress responses), have altered expression of inflammatory responses and higher levels of pathogen immunosuppression. In addition to extensive perturbation of immune pathways, rearing density and infection appear to dysregulate the expression of molecular body clock genes, demonstrating potentially profound and complex effects of rearing conditions that must be considered for managed animal disease mitigation.

*Saprolegnia parasitica* is a generalist pathogen, capable of infecting a broad taxonomic range of freshwater teleosts [21]. To date, transcriptional changes due to infection have only been elucidated in salmonid host species [14, 22, 23]. We found the core responses of tilapia to *Saprolegnia* challenge (i.e. changes in gene expression common to both stocking densities) are broadly similar to those highlighted in salmonid studies; such as early increases in acute inflammation expression including interleukin-mediated skin inflammatory genes (e.g. *il1rl2*, Additional files 5 and 6). The pathogenicity of *Saprolegnia* is likely to be due, at least in part, to its ability to suppress the host’s adaptive immune response [14]. Here, we found evidence for immunosuppression in infected tilapia. In both the skin and gills, there is decreased expression of genes related to adaptive immunity, including T cell and MHC-related genes (Additional files 5, 6, 7 and 8), suggesting the immunosuppressive activities of *Saprolegnia* are not limited to salmonid infections.

There were substantial differences in susceptibility to *Saprolegnia* infection between the two stocking densities, with significantly higher mortality in low density fish (Fig. 1a). We propose underlying differences in expression of genes between the two stocking densities significantly contribute to the observed variance in *Saprolegnia* susceptibility. Although we reveal increased expression of some pro-inflammatory pathways in fish at both densities, early responses unique to low-density fish included increased expression of genes related to downregulation of inflammation (e.g. *tnip1, sgk1, mst1*) in both tissues and a decrease in pro-inflammatory cytokine (interleukin-12, *IL12*) production in the skin (Additional files 5 and 6). In mammals, stress is known as a potent disruptor of pro-inflammatory responses, including glucocorticoid inhibition of *IL12* production [24]. *IL12* is critical to producing Th1 responses and interferon-gamma (*INFγ*) [25], both considered important for clearing *Saprolegnia* and other oomycete infections [18]. Taken alone, these results would suggest that raising tilapia at low stocking density dampens their ability to mount an inflammatory response to *Saprolegnia* challenge, resulting in higher mortality rates. However, low-density fish also exhibited increased expression of interleukin-17 (*IL17*), a pro-inflammatory cytokine produced by Th17 cells [26]. Therefore, low stocking density (and thus perhaps stress) may not universally decrease inflammatory responses, but instead induce an inflammatory pathway less effective against oomycete infections.

In addition to differences in inflammatory responses to *Saprolegnia* between fish held at different stocking densities, we revealed important variation in adaptive immune gene expression. Genes with increased expression in the gills of only high-density (less susceptible) fish were enriched for immunoglobulin production and B cell activation at 24 h. Increased expression of genes such as *batf* and *msh2* suggest immunoglobulin class-switch recombination [27, 28] plays a role in effective defences against *Saprolegnia* infections. T cell mediated immunity was enriched in genes with increased expression at both time points only in high-density fish (Additional file 6). These genes included several transient receptor potential cation channel subfamily M 4 members (e.g. *trpm4*), key to regulating Th responses [29], providing further support for the impact of rearing density on T helper cell activity. While there was evidence of the immunosuppressive activity of *Saprolegnia* in infected fish from both density treatments, these results suggest that fish at high density (with likely lower levels of stress) are less susceptible to adaptive immune pathways disruption, resulting in lower mortality rates. Future work to examine how the gene groups and pathways we identify here are perturbed under various chronic stressors (e.g. temperature, pollutants etc) and other immunosuppressive pathogen species will be highly informative for understanding the impacts of chronic stressors on disease dynamics in both wild and captive animal populations.

Perhaps the most striking finding of this study was the extent to which expression of genes regulating circadian rhythms was affected by stocking density and infection (Fig. 3a). The vast majority of genes central to vertebrate circadian rhythm regulation [30] were differentially expressed between uninfected and infected fish. Genes of both the negative (e.g. *arntl* and *clock*) and positive (e.g. *per* and *cry*) arms of circadian transcriptional loops, plus associated regulators (e.g. *rora*), exhibited decreased expression in infected fish. However, the extent of variation in circadian expression profiles due to infection differed by stocking density, with greater changes observed in fish at low density (Fig. 3b). Moreover, some of the most significant differences in gene expression between stocking densities in unchallenged fish are associated with circadian rhythm functions. In humans and other mammalian models, there is increasing recognition of the intricate links between stress, disease, and circadian rhythms [31]. Despite this, little or no consideration has been given to the implications for managed animal populations. Here, we provide the first indication that, in fish, management practices may substantially impact circadian functioning and thus immunity to pathogens. Time-series of expression during infection (i.e. to determine if circadian gene expression is continually suppressed during infection or expression oscillations are shifted) and further studies under different levels of stress will be highly valuable to our understanding of the impacts of rearing conditions on animal immunity. For aquaculture, where photoperiod manipulation is a common management tool [32, 33], we posit it is essential optimal husbandry conditions are implemented to minimise stress, thus decreasing disease susceptibility and ultimately increasing not only welfare, but economic productivity.

## Conclusions

This study demonstrates the complex functional genomic effects of rearing density on fish immune responses and disease susceptibility. We find that densities that likely induce increased social stress in tilapia result in altered inflammatory responses to *Saprolegnia*, increased immunosuppression of adaptive immune pathways by the pathogen, and thus higher mortality due to disease. By employing a transcriptome-wide approach, we revealed a high level of dysregulation of molecular clock genes due to stress and during infection. Our results provide not only an important in-depth transcriptomic resource for future studies of rearing stress and immunity in fish, but also highlights the need to incorporate circadian infection biology into our understanding of disease dynamics in managed animals.

## Methods

### Tilapia rearing conditions

Four hundred and sixty 3-week-old mixed-sex Nile tilapia fry (standard length 11 to 29 mm, mean = 19.2 mm, weight 0.31 to 4.29 g, mean = 1.54 g) were obtained from a commercial facility (FishGen Ltd.). Fry were visually and microscopically determined free of parasitic infections upon arrival. Fry were randomly allocated to glass aquaria (90 × 30 × 38 cm) and reared for 9 weeks under two stocking conditions. In the low density (LD) treatment, fry (*n* = 94) were maintained at 1.5 kg m^− 3^. In the high density (HD) treatment, fry (*n* = 366) were maintained at 6 kg m^− 3^, thus maintaining 4 times higher density than LD treatment throughout rearing. Both density treatments are within typical fry stocking density regimes in commercial tilapia aquaculture [34]. Fish were fed ad libitum twice daily with trout pellets (Skretting Nutra Parr) and maintained under 12:12 h light: dark conditions at 24 °C (± 0.5 °C) with external filtration. Water quality measurements (ammonia, nitrite, nitrate) and 50% water changes were performed every other day in both treatments to ensure equivalent abiotic conditions (ammonia, nitrite = 0 ppm, nitrate = 20–30 ppm).

### Experimental infections

*Saprolegnia parasitica* (species confirmed with rDNA ITS sequencing according to [35], isolated in 2015 from a naturally infected *Salmo salar*), were used for experimental infections. Mycelial cultures were maintained at 24 °C on potato dextrose agar plates. Prior to zoospore production, mycelial plugs were transferred to glucose yeast extract broth and incubated for 3 days at 24 °C. Mycelia masses were washed in sterile dechlorinated water and then maintained for 3 days in 50% dechlorinated water, 50% water from tilapia stock tanks, at 15 °C to induce zoospore production. Heat-killed zoospores were produced by incubating at 60 °C for 1 h. A haemocytometer was used to quantify zoospore concentrations and zoospore suspensions were equilibrated to 24 °C before use [36].

In both density conditions, fish were divided into challenged (HD; *n* = 285, LD; *n* = 73) and sham-challenged control (HD; *n* = 80, LD; *n* = 20) groups, maintaining 1.5 kg m^− 3^ and 6 kg m^− 3^ in low- and high-density treatments respectively. The sample size for *Saprolegnia*-challenged groups was based on previous *Saprolegnia* differential susceptibility experiments using other stressors [37]. To avoid the overriding effects of acute stress due to confinement and individual isolation [38], fish were simultaneously challenged with *S. parasitica* in these treatment groups. Immediately prior to exposure, all fish were shaken in a hand net in groups of five for 1 min to facilitate infection [39]. In the high-density treatment, live zoospores were added directly to aquaria to achieve a concentration of 5 × 10^6^ zoospores L^− 1^. To ensure low density fish were exposed to equivalent 1) number of infective zoospores per individual and 2) concentration of organic matter, a mixture of 1:3 live: heat-killed zoospores was added directly to each aquarium to achieve a concentration of 5 × 10^6^ zoospores L^− 1^. Filtration was turned off during exposure and zoospore solutions were completely changed in all tanks every 6 h to minimise waste accumulation. For uninfected controls, sham-challenged fish were also net-shaken, returned to holding aquaria, and water changed every 6 h to mimic the *Saprolegnia*-exposed groups. After 24 h, water was completely changed in all tanks and filtration turned back on.

For the 24-h exposure period and duration of infection experiment, fish were visually inspected hourly (under red light during dark periods) for signs of lethargy, reduced swimming ability and mycelial growth. Fish showing severe signs of infection were immediately euthanised with an overdose of Tricaine Methanesulfonate (MS222, 500 mg L^− 1^) according to Home Office Schedule 1. All fish were weighed, standard length measured, and body condition calculated (Fulton’s Condition Index; K = weight/standard-length^3^). At 24 and 48 h post-exposure, five fish per treatment combination (high/low density, challenged/sham-challenged) were euthanised with an overdose of MS222 (500 mg L^− 1^) and samples of gill (all arches) and skin (standardised right and left flank area immediately posterior to opercula) were immediately preserved in RNAlater and stored at − 80 °C until RNA extraction. Skin and gill tissues were chosen as they are primary sites of infection of *Saprolegnia*. Individuals selected for gene expression analyses were size-matched among density and infection treatments. For infected fish, to ensure gene expression signals were not simply due severe physiological stress near death, individuals were selected showing mycelial growth but no other signs of pathology (e.g. loss of swimming/righting ability, lethargy, hypo/hyperventilation). All tissue samples used for gene expression analyses were taken at the same time of day (11.00 am). In the absence of reliable methods to quantify *Saprolegnia* infection burdens, here we refer to disease “susceptibility/resistance” as mortality/survival post-challenge. Statistical analyses of survival were performed in R. The effect of stocking density, fish size, and body condition on *Saprolegnia* challenge survival was examined using Cox proportional hazards (R package survival [40]). Continuous variables (length, condition) were scaled and centred on the mean. The initial model predicted survival using all variables (density treatment, length, condition) and their two-way interactions. Stepwise model selection on Akaike information criterion (AIC) was then performed. The interaction of density and length on probability of survival was further examined using plots of binary logistic regressions.

### Transcriptome sequencing

Total RNA was extracted from each tissue sample separately using AllPrep DNA/RNA Micro kit (Qiagen). RNA was quantified using Qubit High-Sensitivity RNA assays (ThermoFisher Scientific) and integrity was determined using Agilent 4200 Tapestation RNA assays (Agilent Technologies). All samples had RNA integrity values greater than 8.0. Libraries were generated using the Illumina Stranded TruSeq mRNA sample preparation kits (high-throughput protocol), as per the manufacturer’s instructions (Illumina, San Diego, CA). Libraries’ quality was quantified and assessed using Agilent 4200 Tapestation prior equimolar pooling. The library pool was run four times on Illumina NextSeq500 (2 × 75 PE) to achieve a minimum of 20 million read pairs per sample. Raw reads are available at the NCBI Short Read Archive under Accession Number PRJNA413738.

### Gene expression analyses

Reads were filtered and trimmed using Trimmomatic version 0.32 [41] to: (1) trim any Illumina adapter sequence, (2) trim the 5′ and/or 3′ end of reads where quality score dropped below Q20, (3) trim anywhere within each read where a 5-bp window drops below Q20, and (4) discard any trimmed reads less than 36 bp long. This ensured only the highest quality reads were used for subsequent analyses. Trimmed reads were then mapped to the *O. niloticus* genome (v1.1 [42]) using HISAT2 version 2.0.5 [43] and quantified using RSEM version 1.2.30 [44]. A summary of sequence read data and mapping is presented in Table 1. Gene expression profiles were visualised using principal component analysis (PCA) plots of z-scaled and centred read counts to ensure no individual outliers driving expression differences [45] (Additional file 12). Differential expression tests were performed using the R package DESeq2 [46], comparing 1) high- and low-density control (uninfected) fish at both time points (24 and 48 h post-exposure), and 2) infected and uninfected (control) fish at both time points (24 and 48 h post-exposure). Transcripts were filtered to include only those with at least two counts per million mapped reads (TPM) in at least two individuals. Although fish were size matched between groups, as fish size was significant in models of survival, length was included as a covariate in expression testing and tissues were analysed separately. False discovery rate (FDR) corrected *P* values of less than 0.05 were considered for significant differential expression. Overlap of differentially expressed genes between treatment groups were determined using Venny version 2.1 [47]. Gene ontology (GO) functional enrichment tests (with FDR correction) were carried out via Blast2GO [48] to detect significantly overrepresented biological processes, molecular functions, and cellular components of groups of differentially expressed genes shared/unique to particular treatment groups.

**Table 1:** Summary of *Oreochromis niloticus* transcriptome sequence data

Sample ID	Tissue	Treatment	Total raw read pairs (M)	Average raw quality score	Cleaned uniquely mapped read pairs (M)
HD18-G	Gill	HD24	24.09	34.05	17.72
HD19-G	Gill	HD24	24.66	34.06	18.08
HD20-G	Gill	HD24	25.72	34.01	18.8
HD21-G	Gill	HD24	29.76	33.94	21.2
HD23-G	Gill	HD24	26.95	34.11	19.42
HD24-G	Gill	HD48	26.44	33.94	18.93
HD25-G	Gill	HD48	24.84	34.03	18.38
HD26-G	Gill	HD48	24.47	34.02	17.85
HD27-G	Gill	HD48	25.01	33.93	17.42
HD28-G	Gill	HD48	23.43	34.03	17.23
HDC10-G	Gill	HDC24	23.36	34.06	16.99
HDC6-G	Gill	HDC24	26.95	34.01	19.4
HDC7-G	Gill	HDC24	25.48	34.06	18.65
HDC8-G	Gill	HDC24	25.72	33.87	17.96
HDC9-G	Gill	HDC24	24.13	34.02	17.37
HDC11-G	Gill	HDC48	26.36	34.03	18.69
HDC12-G	Gill	HDC48	24.00	34.06	17.73
HDC13-G	Gill	HDC48	25.20	34.04	18.02
HDC14-G	Gill	HDC48	25.42	33.75	18.51
HDC15-G	Gill	HDC48	24.83	34.03	18.09
LD28-G	Gill	LD24	25.17	34.09	18.45
LD29-G	Gill	LD24	25.65	33.99	18.24
LD30-G	Gill	LD24	26.78	34.06	19.89
LD31-G	Gill	LD24	25.46	33.98	18.61
LD32-G	Gill	LD24	25.32	33.39	18.43
LD35-G	Gill	LD48	26.99	33.99	19.82
LD36-G	Gill	LD48	25.37	34.03	18.63
LD37-G	Gill	LD48	26.28	33.70	19.2
LD38-G	Gill	LD48	28.75	34.03	21.1
LD39-G	Gill	LD48	23.87	33.94	17.41
LDC10-G	Gill	LDC24	28.68	34.06	20.96
LDC6-G	Gill	LDC24	25.97	34.08	19.15
LDC7-G	Gill	LDC24	25.69	33.88	18.59
LDC8-G	Gill	LDC24	25.99	34.08	19.07
LDC9-G	Gill	LDC24	23.15	33.97	16.86
LDC11-G	Gill	LDC48	30.69	33.99	22.33
LDC12-G	Gill	LDC48	26.03	33.95	18.82
LDC13-G	Gill	LDC48	25.36	33.89	18.23
LDC14-G	Gill	LDC48	26.60	34.03	19.28
LDC15-G	Gill	LDC48	26.98	33.79	19.19
HD18-SK	Skin	HD24	25.74	33.55	18.85
HD19-SK	Skin	HD24	24.29	33.95	18.22
HD20-SK	Skin	HD24	25.23	33.90	18.98
HD21-SK	Skin	HD24	32.66	33.95	24.31
HD23-SK	Skin	HD24	24.46	33.95	18.43
HD24-SK	Skin	HD48	23.52	33.82	17.29
HD25-SK	Skin	HD48	24.95	33.90	18.49
HD26-SK	Skin	HD48	24.40	34.01	18.67
HD27-SK	Skin	HD48	24.86	33.85	17.71
HD28-SK	Skin	HD48	23.02	33.91	17.14
HDC10-SK	Skin	HDC24	24.51	33.89	18.09
HDC6-SK	Skin	HDC24	26.77	33.95	19.59
HDC7-SK	Skin	HDC24	24.29	33.99	18.05
HDC8-SK	Skin	HDC24	26.79	33.90	18.97
HDC9-SK	Skin	HDC24	27.00	33.99	20.34
HDC11-SK	Skin	HDC48	26.47	33.87	18.53
HDC12-SK	Skin	HDC48	20.60	33.88	15.28
HDC13-SK	Skin	HDC48	23.63	33.82	17.51
HDC14-SK	Skin	HDC48	23.52	33.73	17.2
HDC15-SK	Skin	HDC48	25.09	33.88	18.38
LD28-SK	Skin	LD24	24.84	34.04	18.65
LD29-SK	Skin	LD24	25.10	33.93	18.08
LD30-SK	Skin	LD24	25.33	34.03	19.42
LD31-SK	Skin	LD24	23.97	34.07	18.2
LD32-SK	Skin	LD24	26.73	33.78	20.28
LD35-SK	Skin	LD48	22.38	34.02	17.2
LD36-SK	Skin	LD48	24.52	33.94	18.2
LD37-SK	Skin	LD48	26.56	33.94	20.19
LD38-SK	Skin	LD48	25.28	33.99	19.32
LD39-SK	Skin	LD48	25.97	33.87	19.69
LDC10-SK	Skin	LDC24	25.14	33.99	18.95
LDC6-SK	Skin	LDC24	24.14	34.01	18.12
LDC7-SK	Skin	LDC24	25.76	33.79	18.87
LDC8-SK	Skin	LDC24	25.81	33.99	19.23
LDC9-SK	Skin	LDC24	26.03	33.97	19.29
LDC11-SK	Skin	LDC48	25.62	34.04	19.09
LDC12-SK	Skin	LDC48	26.02	33.95	19.55
LDC13-SK	Skin	LDC48	30.93	34.03	23.1
LDC14-SK	Skin	LDC48	25.60	34.05	19.46
LDC15-SK	Skin	LDC48	26.13	33.96	18.07

(Treatment codes: *HD* high density, *LD* low density, *C* sham-challenge control, 24 24 h post-exposure, 48 48 h post-exposure)

### Weighted gene co-expression network analyses

Differential gene expression (DGE) is usually identified using exact tests carried out on each gene separately; however, due to the need of correcting *p*-values with stringent multiple testing methods, only genes with the largest differences in expression are typically identified. An alternative for quantifying transcriptional responses is weighted gene co-expression network analysis (WGCNA [49]). This method was used to quantify transcriptional responses of fish to stocking density and infection challenge by *Saprolegnia*, enabling the identification of networks (modules) of co-expressed genes (i.e. genes that show consistent expression profiles across samples), and thus potentially identifying functionally important genes with only subtle changes in expression that may otherwise (i.e. via DGE) not have be detected. Trimmed mean of M-values (TMM) normalised fragments per kilobase per million mapped expression values were analysed using the R package WGCNA [49]. Our gene modules were defined using the dynamicCutTree function and TOMType “signed” with a minimum module size of 100. A module eigengene distance threshold of 0.25 was used to merge highly similar modules. Modules were considered robust if the average module adjacencies were significantly higher than observed in a set of 1000 random module permutations. Modules were then correlated with time, stocking density, infection and fish length to identify gene networks significantly associated with factors of interest. GO term enrichment tests of each significant gene module were performed using Blast2Go as described above. Each gene within a module was ranked by its module membership (kME), calculated by WGCNA. Network hub genes were defined as those ranked in the top 100 module membership values and with the highest 150 network connection weights. Hub gene network connections were exported to Cytoscape [50] for visualization.

## Additional files

Additional file 1: Cox Proportional Hazards model selection: Results of Cox Proportional Hazards models for evaluating the effect of density on survival during *Saprolegnia* infection, showing the model selection process for the best fit of the data according to AIC values. (DOCX 14 kb)

Additional file 2: Gene Ontology enrichment of uninfected gill tissues: Full GO enrichment results of gill genes differentially expressed between uninfected fish at high and low density. (XLSX 6567 kb)

Additional file 3: Gene Ontology enrichment of uninfected skin tissues: Full GO enrichment results of skin genes differentially expressed between uninfected fish at high and low density. (XLSX 3920 kb)

Additional file 4: Gene Ontology enrichment of skin gene modules: Full GO enrichment results of gill gene co-expression modules. (PDF 80 kb)

Additional file 5: Summary of skin gene ontology enrichment: Summary of gene ontology (GO) term enrichment of skin genes differentially expressed between control (uninfected) and *Saprolegnia*-infected *Oreochromis niloticus* density treatment groups (LD; low density, HD; high density), including total number of differentially expressed genes, most significant GO term, and major biological process clusters determined using ReViGO. **↑** denotes increased expression in infected fish and **↓** denotes decreased expression. (DOCX 38 kb)

Additional file 6: Summary of gill gene ontology enrichment: Summary of gene ontology (GO) term enrichment of gill genes differentially expressed between control (uninfected) and *Saprolegnia*-infected *Oreochromis niloticus* density treatment groups (LD; low density, HD; high density), including total number of differentially expressed genes, most significant GO term, major biological process clusters determined using ReViGO. ↑ denotes increased expression in infected fish and ↓ denotes decreased expression. (XLSX 2288 kb)

Additional file 7: Summary of skin gene module enrichment: Summary of gene ontology (GO) term enrichment of skin gene co-expression modules defined by WGCNA, including total number of genes per module, most significant GO term, and major biological process clusters determined using ReViGO. Significant correlations with infection status, density treatment, time point, and fish standard length indicated (+; positive correlation, −; negative correlation). (XLSX 2803 kb)

Additional file 8: Summary of gill gene module enrichment: Summary of gene ontology (GO) term enrichment of gill gene co-expression modules defined by WGCNA, including total number of genes per module, most significant GO term, and major biological process clusters determined using ReViGO. Significant correlations with infection status, density treatment, time point, and fish standard length indicated (+; positive correlation, −; negative correlation). (XLSX 5834 kb)

Additional file 9: Gene Ontology enrichment of infected gill tissues: Full GO enrichment results of gill genes differentially expressed between infected and uninfected fish at high and low density. (DOCX 21 kb)

Additional file 10: Gene Ontology enrichment of infected skin tissues: Full GO enrichment results of skin genes differentially expressed between infected and uninfected fish at high and low density. (DOCX 21 kb)

Additional file 11: Gene Ontology enrichment of gill gene modules: Full GO enrichment results of gill gene co-expression modules. (DOCX 17 kb)

Additional file 12: Principal Component Analysis plots of gene expression: Principal components analysis (PCA) plots of total gene expression profiles in A) gill, and B) skin of uninfected (CTL) and *Saprolegnia*-challenged (INF) *Oreochormis niloticus* at two stocking densities (H; high, L; low) and time points (24; 24 h post-exposure, 48; 48 h). (DOCX 16 kb)

Additional file 13: Dot plots of GO term enrichment: Summary of GO term enrichment in differentially expressed genes comparing uninfected and *Saprolegnia*-challenged fish. Dot colour intensity indicates FDR-corrected *P* value, dot size indicates number of genes. (XLSX 7015 kb)

## Abbreviations

AIC: Akaike information criterion
bp: Base pair
DGE: Differential gene expression
FDR: False discovery rate
GO: Gene ontology
HD: High-density
ITS: Internal transcribed spacer
LD: Low-density
MHC: Major histocompatibility complex
MS222: Tricaine Methanesulfonate
PCA: Principal component analysis
RNAseq: Whole-transcriptome RNA sequencing
Th: T helper
TMM: Trimmed mean of M-values
WGCNA: Weight gene co-expression network analysis
