# Supplementary material for: Transcriptomic response to parasite infection in Nile tilapia (Oreochromis niloticus) depends on rearing density
Source: BMC Genomics. 2018 Oct 1;19:723. doi: 10.1186/s12864-018-5098-7 (PMC6167859; doi:10.1186/s12864-018-5098-7)
Supplement: Supplementary file 1 — Cox Proportional Hazards model selection: Results of Cox Proportional Hazards models for evaluating the effect of density on survival during Saprolegnia infection, showing the model selection process for the best fit of the data according to AIC values. (DOCX 14 kb) [file 12864_2018_5098_MOESM1_ESM.docx]

**Table S1**. Results of Cox Proportional Hazards models for evaluating the effect of density on survival during *Saprolegnia* infection, showing the model selection process for the best fit of the data according to AIC values.

|  | *coef* | *z* | *P* |
| --- | --- | --- | --- |
| **a)** |  |  |  |
| Density | 3.355 | 8.267 | <2e-16 |
| Body condition | 0.104 | 0.300 | 0.765 |
| Length | 3.151 | 5.569 | 2.56e-8 |
| Density x Condition | -0.873 | -1.415 | 0.157 |
| Density x Length | -1.892 | -2.638 | 0.008 |
| *AIC = 666.38* |  |  |  |
| **b)** |  |  |  |
| Density | 3.280 | 8.111 | 5.55e-16 |
| Body condition | -0.165 | -0.576 | 0.564 |
| Length | 3.116 | 5.560 | 2.69e-8 |
| Density x Length | -1.564 | -2.299 | 0.022 |
| *AIC = 666.37* |  |  |  |
| **c)** |  |  |  |
| Density | 3.246 | 8.097 | 5.55e-16 |
| Length | 3.136 | 5.578 | 2.44e-8 |
| Density x Length | -1.508 | -2.221 | 0.026 |
| *AIC = 664.70* |  |  |  |
