# Supplementary figures and images for: Transcriptomic response to parasite infection in Nile tilapia (Oreochromis niloticus) depends on rearing density
Source: BMC Genomics. 2018 Oct 1;19:723. doi: 10.1186/s12864-018-5098-7 (PMC6167859; doi:10.1186/s12864-018-5098-7)

A

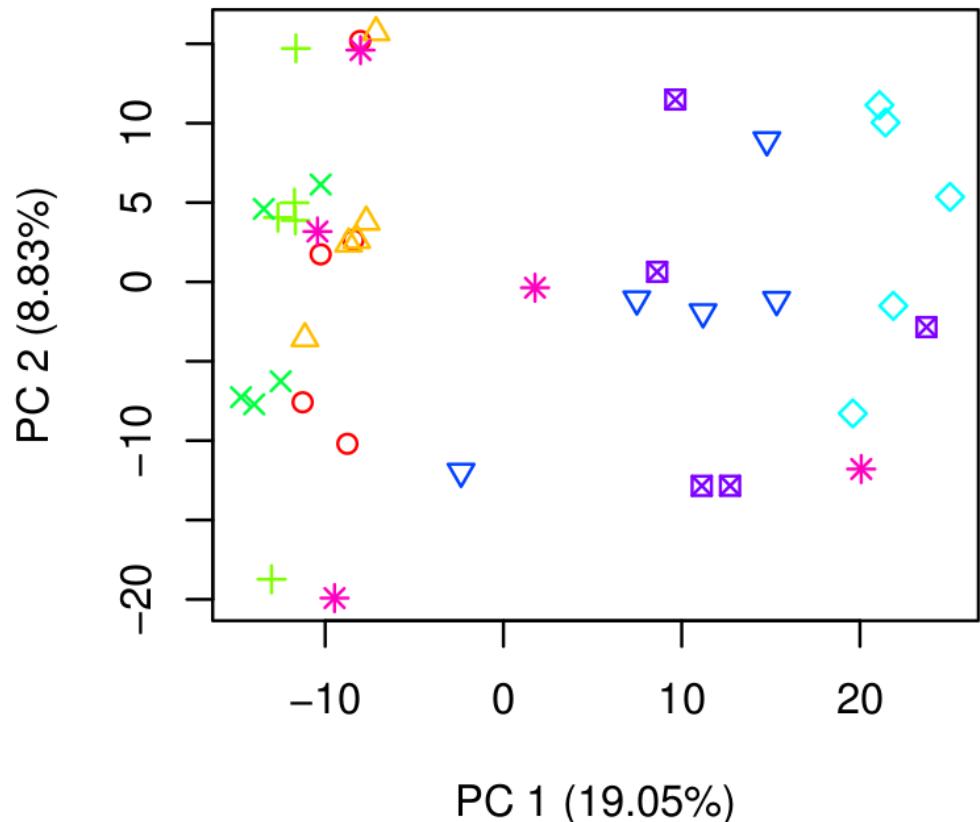

B

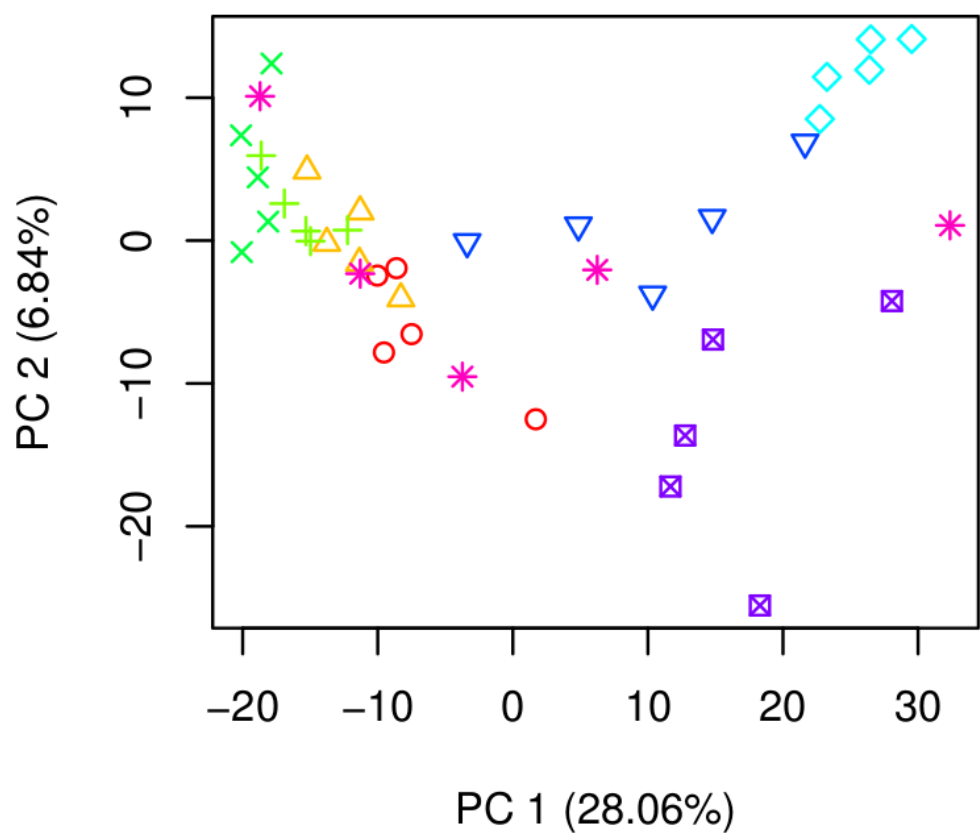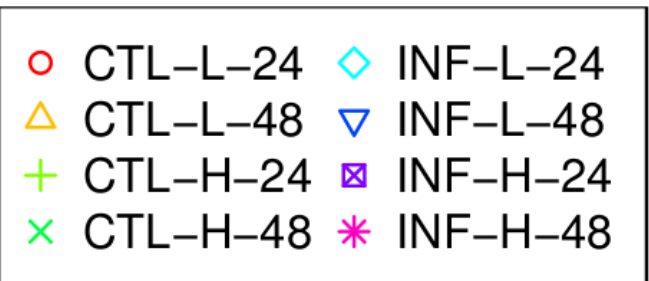

Supplement: Supplementary file 4 — Gene Ontology enrichment of skin gene modules: Full GO enrichment results of gill gene co-expression modules. (PDF 80 kb) [file 12864_2018_5098_MOESM4_ESM.pdf]
