# Supplementary material for: Transcriptomic response to parasite infection in Nile tilapia (Oreochromis niloticus) depends on rearing density
Source: BMC Genomics. 2018 Oct 1;19:723. doi: 10.1186/s12864-018-5098-7 (PMC6167859; doi:10.1186/s12864-018-5098-7)
Supplement: Supplementary file 9 — Gene Ontology enrichment of infected gill tissues: Full GO enrichment results of gill genes differentially expressed between infected and uninfected fish at high and low density. (DOCX 21 kb) [file 12864_2018_5098_MOESM9_ESM.docx]

**Table ST2.** Summary of gene ontology (GO) term enrichment of skin genes differentially expressed between control (uninfected) and *Saprolegnia*-infected *Oreochromis niloticus* density treatment groups (LD; low density, HD; high density), including total number of differentially expressed genes, most significant GO term, and major biological process clusters determined using ReViGO. **↑** denotes increased expression in infected fish and **↓** denotes decreased expression.

| **Expression** | | | | **No. genes** | **Top GO** | **ReViGO groups** | **Infection & stress related terms** |
| --- | --- | --- | --- | --- | --- | --- | --- |
| 24 hr | | 48 hr | |  |  |  |  |
| LD | HD | LD | HD |  |  |  |  |
| **↑** |  |  |  | 1,965 | Protein phosphorylation | Intracellular signal transduction, protein phosphorylation, vesicle-mediated transport, actin cytoskeleton organization | Inflammatory response, I-kappaB kinase/NF-kappaB signalling, viral entry into host cell, MyD88-dependent toll-like receptor signalling pathway |
|  | **↑** |  |  | 905 | Small molecule biosynthetic process | Carboxylic acide metabolic process, glucose 6-phosphate metabolism, tRNA metabolism, angioblast cell migration | Metallopeptidase activity |
|  |  | **↑** |  | 1,003 | RNA processing | RNA processing, organophosphate biosynthesis, intracellular transport, macromolecular complex subunit organization | Positive regulation of I-kappaB kinase/NF-kappaB signalling, myeloid leukocyte migration, response to fungus, cortisol secretion |
|  |  |  | **↑** | 108 | ARF protein signal transduction | Regulation of ARF protein signal transduction, podosome assembly, bone development, pigment granule aggregation in cell centre | N/A |
| **↑** | **↑** |  |  | 990 | D-gluconate catabolic process | Muscle attachment, clathrin-mediated endocytosis, aldonic acid catabolism, TOR signalling | Mast cell migration, leukocyte differentiation, positive regulation of nitric oxide metabolic process, wound healing, epithelial cell migration, interleukin-1 receptor activity, |
|  |  | **↑** | **↑** | 15 | Regulation of mitotic cell cycle, embryonic | Mitotic cell cycle | N/A |
| **↑** |  | **↑** |  | 369 | Cellular lipid metabolic process | Cellular lipid metabolic process, macromolecule localization, wound healing, regulation of transferase activity | Wound healing, response to stress, chemokine activity, defence response, tissue regeneration |
|  | **↑** |  | **↑** | 51 | Negative regulation of multicellular organismal process | Negative regulation of multicellular organismal process, negative chemotaxis | Chemorepellant activity, blood vessel morphogenesis |
|  | **↑** | **↑** |  | 691 | RNA processing | RNA processing, ribonucleoprotein complex biogenesis, protein localization, antigen processing and presentation | Antigen processing and presentation of exogenous peptide antigen via MHC class II, response to stress, immune system development, viral process, leukotriene-A4 hydrolase activity, type I interferon production |
| **↑** |  |  | **↑** | 55 | Myotome development | Regulation of myotome development, histone H3 deacetylation, cell-cell adhesion, pigmentation | N/A |
| **↑** | **↑** | **↑** |  | 868 | Catabolic process | Protein catabolism, peptide metabolism, hexose metabolism, ribonucleoprotein complex biogenesis | Response to oxidative stress, Fc receptor signalling pathway, immune response-activating cell surface receptor signalling pathway, leukotriene B4 receptor activity, mucus secretion, response to stress, |
| **↑** |  | **↑** | **↑** | 23 | Regulation of endocytosis | Regulation of endocytosis, biological regulation, actin filament based process | Stress-activated MAPK cascade |
| **↑** | **↑** |  | **↑** | 150 | Small GTPase mediated signal transduction | Small GTPase mediated signal transduction, hemidesmosome assembly, lipid digestion, circadian rhythm | Response to transforming growth factor beta |
|  | **↑** | **↑** | **↑** | 21 | Fatty acid biosynthetic process | Fatty acid biosynthetic process | N/A |
| **↑** | **↑** | **↑** | **↑** | 148 | Response to methanol | Cellular response to methanol, lipid localization, long-chain fatty acid metabolism, oxidation-reduction process | N/A |
| **↓** |  |  |  | 1,986 | Cell cycle | DNA replication, cell cycle, chromosome organization, cellular metabolism | C-X-C chemokine receptor activity, regulation of leukocyte migration, NIK/NF-kappaB signalling, T cell receptor complex, B cell mediated immunity, immunoglobulin mediated immune response, inflammatory response |
|  | **↓** |  |  | 1,332 | Dense core granule exocytosis | Intracellular signal transduction, production of molecular mediator of immune response, actin filament organization, dense core granule exocytosis | T cell mediated immunity, cytokine production involved in immune response, antibiotic transporter activity, apoptotic process |
|  |  | **↓** |  | 796 | Negative regulation of extrinsic apoptotic signalling pathway | Apoptotic signalling pathway, epithelial structure maintenance | Epidermis morphogenesis, circadian rhythm |
|  |  |  | **↓** | 81 | Bicarbonate transport | Bicarbonate transport, actomyosin structure organization, fructose 2,6-biphosphate metabolism, pyruvate metabolism | N/A |
| **↓** | **↓** |  |  | 835 | Protein phosphorylation | Negative regulation of growth, liver development, protein phosphorylation, endoplasmic reticulum organization | Gland development, fibroblast growth factor receptor signalling pathway, intermediate filament-based process, regulation of granulocyte differentiation |
|  |  | **↓** | **↓** | 15 | Response to transforming growth factor beta | Transforming growth factor beta receptor signalling pathway | Transforming growth factor beta receptor signalling pathway |
| **↓** |  | **↓** |  | 447 | Reelin-mediated signalling pathway | Reelin-mediated signalling pathway, neuronal ion channel clustering, taurine transport, peptidyl-proline hydroxylation | Collagen biosynthetic process |
|  | **↓** |  | **↓** | 84 | Alcohol metabolic process | Alcohol metabolic process, glycerol metabolic process, alditol metabolic process, cellular lipid metabolic process | N/A |
|  | **↓** | **↓** |  | 444 | Retrograde axonal transport | Regulation of JNK cascade, axon cargo transport, skin development, cell communication | Stress-activated protein kinase signalling cascade, regulation of cellular response to stress, leukocyte migration involved in inflammatory response, interleukin-12 production, macrophage chemotaxis, metallocarboxypeptidase activity |
| **↓** |  |  | **↓** | 40 | N/A | N/A | N/A |
| **↓** | **↓** | **↓** |  | 939 | Tyrosine metabolic process | Cell surface receptor signalling pathway, tyrosine metabolism, protein dephosphorylation, cell adhesion | MHC class II protein complex, positive regulation of T cell differentiation, T cell activation, leukocyte cell-cell adhesion, fin regeneration, cellular response to corticosteroid stimulus |
| **↓** |  | **↓** | **↓** | 9 | N/A | N/A | N/A |
| **↓** | **↓** |  | **↓** | 45 | Pronephros development | Pronephros development, antigen processing and presentation, Wnt signalling pathway, cobalamin transport | MHC class II protein complex, antigen processing and presentation of peptide or polysaccharide antigen via MHC class I, immune response |
|  | **↓** | **↓** | **↓** | 45 | N/A | N/A | N/A |
| **↓** | **↓** | **↓** | **↓** | 75 | Immune response | Immune response, immune system process, fructose metabolism, signalling | Interferon-gamma-mediated signalling pathway |
