# Supplementary material for: Transcriptomic response to parasite infection in Nile tilapia (Oreochromis niloticus) depends on rearing density
Source: BMC Genomics. 2018 Oct 1;19:723. doi: 10.1186/s12864-018-5098-7 (PMC6167859; doi:10.1186/s12864-018-5098-7)
Supplement: Supplementary file 10 — Gene Ontology enrichment of infected skin tissues: Full GO enrichment results of skin genes differentially expressed between infected and uninfected fish at high and low density. (DOCX 21 kb) [file 12864_2018_5098_MOESM10_ESM.docx]

**Table ST3.** Summary of gene ontology (GO) term enrichment of gill genes differentially expressed between control (uninfected) and *Saprolegnia*-infected *Oreochromis niloticus* density treatment groups (LD; low density, HD; high density), including total number of differentially expressed genes, most significant GO term, major biological process clusters determined using ReViGO. **↑** denotes increased expression in infected fish and **↓** denotes decreased expression.

| **Expression** | | | | **No. genes** | **Top GO** | **ReViGO groups** | **Infection & stress related terms** |
| --- | --- | --- | --- | --- | --- | --- | --- |
| 24 hr | | 48 hr | |  |  |  |  |
| LD | HD | LD | HD |  |  |  |  |
| **↑** |  |  |  | 1,911 | Cilium morphogenesis | Cilium morphogenesis, regulation of inflammatory response, cellular protein modification process, protein complex localization | Transforming growth factor beta-activated receptor activity, inflammatory response, mast cell migration, leukotriene-C4 synthase activity, response to oxidative stress, skin epidermis development |
|  | **↑** |  |  | 914 | DNA replication | DNA replication, cell cycle process, chromosome organization, angioblast migration | Immunoglobulin production involved in immunoglobulin mediated immune response, response to stress, B cell activation involved in immune response, chemokine binding |
|  |  | **↑** |  | 436 | Cofactor metabolic process | Small molecule metabolism, phospholipid translocation, mRNA modification, coenzyme metabolism | N/A |
|  |  |  | **↑** | 92 | Mitotic cell cycle | Regulation of hormone levels, mitotic cell cycle, response to xenobiotic stimulus, chromatin remodelling | Cellular response to xenobiotic stimulus |
| **↑** | **↑** |  |  | 1,078 | Cellular nitrogen compound metabolic process | NcRNA metabolism, response to stress, ribonucleoprotein complex biogenesis, epidermis morphogenesis | Epidermis morphogenesis, interleukin-4 binding, response to osmotic stress, viral entry into host cell, response to fungus, mucus secretion, response to bacterium |
|  |  | **↑** | **↑** | 23 | Histone H3-K4 demethylation | Vitamin metabolism, regulation of isoprenoid metabolism, histone H3-H4 demethylation, monocarboxylic acid transport | Regulation of coagulation |
| **↑** |  | **↑** |  | 246 | Regulation of kinase activity | Regulation of phosphate metabolism, cardiovascular system development, glutamate biosynthesis, actin crosslink formation | Blood vessel morphogenesis, virion attachment to host cell, antigen processing and presentation of peptide or polysaccharide antigen via MHC class II, response to yeast |
|  | **↑** |  | **↑** | 64 | Cell cycle process | Cell cycle, T cell cytokine production, circulatory system process, nuclear division | T cell mediated immunity, adaptive immune response, T cell cytokine production, immune effector process |
|  | **↑** | **↑** |  | 416 | Mitochondrion organization | RNA processing, intracellular transport, glucose metabolism, mitochondrion organization | Regulation of circadian rhythm |
| **↑** |  |  | **↑** | 38 | Vitamin transmembrane transport | Vitamin transmembrane transport, triglyceride homeostasis | Fc receptor signalling pathway, immune response-regulating signalling pathway |
| **↑** | **↑** | **↑** |  | 968 | Organonitrogen compound biosynthetic process | NcRNA metabolism, ribosome biogenesis, lipid metabolism, peripheral nervous system development | Response to stress, leukotriene metabolic process, leukotriene B4 receptor activity, B cell mediated immunity, immunoglobulin mediated immune response |
| **↑** |  | **↑** | **↑** | 30 | Peripheral nervous system myelin maintenance | Myelin maintenance, mast cell activation | Mast cell activation, myeloid leukocyte activation |
| **↑** | **↑** |  | **↑** | 74 | Proteolysis | Nucleotide sugar metabolism, proteolysis, metabolism, chondrocyte development | N/A |
|  | **↑** | **↑** | **↑** | 35 | Carbohydrate derivative metabolic process | Fructose catabolism, monocarboxylic acid metabolism, carbohydrate derivative metabolism, carbon utilization | N/A |
| **↑** | **↑** | **↑** | **↑** | 175 | Single-organism metabolic process | Lipid biosynthesis, macromolecule catabolism, ER-nucleus signalling pathway, coenzyme metabolism | Antibiotic metabolic process |
| **↓** |  |  |  | 1,667 | Polysaccharide metabolic process | Polysaccharide metabolism, protein kinase C-activating G-protein coupled receptor signalling pathway, phospholipid metabolism, cytoplasmic transport | Antibiotic transport, interleukin-12 production, antigen processing and presentation of exogenous peptide antigen via MHC class II, |
|  | **↓** |  |  | 975 | Regulation of intracellular signal transduction | Regulation of intracellular signal transduction, circulatory system development, inositol metabolism, dense core granule exocytosis | Metallocarboxypeptidase activity, positive regulation of stress-activated MAPK cascade, xenophagy, epithelium migration |
|  |  | **↓** |  | 661 | Regulation of striated muscle contraction | Regulation of striated muscle contraction, plasma membrane organization, mast cell migration, self proteolysis | Regulation of circadian rhythm, mast cell migration, cytokine activity, regulation of myeloid leukocyte differentiation, apoptotic process, immune response, tumor necrosis factor receptor binding |
|  |  |  | **↓** | 78 | Myotome development | Smoothened signalling pathway, blood circulation, cell communication | Skin morphogenesis, immune response, blood vessel development |
| **↓** | **↓** |  |  | 1,214 | Cell surface receptor signalling pathway | Cell surface receptor signalling pathway, circulatory system development, protein phosphorylation, ameboidal-type cell migration | Corticosteroid receptor signalling pathway, keratin filament |
|  |  | **↓** | **↓** | 45 | N/A | N/A | N/A |
| **↓** |  | **↓** |  | 281 | Cortical actin cytoskeleton organization | Bulbus arterosis formation, complement activation, cortical actin cytoskeleton organization, cellular amino acid catabolism | Complement activation, MHC class II protein complex, humoral immune response |
|  | **↓** |  | **↓** | 41 | Cell migration | Cell motility, regulation of neuronal synaptic plasticity, cytoskeleton organization | N/A |
|  | **↓** | **↓** |  | 281 | Skeletal myofibril assembly | Cellular response to carbohydrate stimulus, skeletal myofibril assembly, cytokine metabolism | Interleukin-12 production, NF-kappaB complex |
| **↓** |  |  | **↓** | 45 | Regulation of cell adhesion | Organ maturation, regulation of cell adhesion, phospholipid catabolism | Negative regulation of T cell activation |
| **↓** | **↓** | **↓** |  | 785 | Retrograde axonal transport | Regulation of JNK cascade, retrograde axon cargo transport, extracellular matrix organization | Regulation of stress-activated MAPK cascade, collagen fibril organization, chemokine activity, positive regulation of I-kappaB kinase/NF-kappaB signalling, immune system development, MHC protein binding |
| **↓** |  | **↓** | **↓** | 30 | N/A | N/A | N/A |
| **↓** | **↓** |  | **↓** | 69 | Calcium-dependent cell-cell adhesion via plasma membrane cell adhesion molecules | Negative regulation of cellular process, calcium ion import into cytosol, nucleic acid-templated transcription | Lymphangiogenesis, immune system process, response to wounding, B cell receptor signalling pathway, epithelium development, T cell receptor signalling pathway |
|  | **↓** | **↓** | **↓** | 27 | Regulation of transcription, DNA-templated | Regulation of transcription, actin cytoskeleton reorganization | N/A |
| **↓** | **↓** | **↓** | **↓** | 105 | Photoperiodism | Circadian rhythm, zymogen activation, regulation of cell shape | Photoperiodism, circadian rhythm, collagen biosynthetic process, MHC class II protein binding |
