# Supplementary material for: Transcriptomic response to parasite infection in Nile tilapia (Oreochromis niloticus) depends on rearing density
Source: BMC Genomics. 2018 Oct 1;19:723. doi: 10.1186/s12864-018-5098-7 (PMC6167859; doi:10.1186/s12864-018-5098-7)
Supplement: Supplementary file 11 — Gene Ontology enrichment of gill gene modules: Full GO enrichment results of gill gene co-expression modules. (DOCX 17 kb) [file 12864_2018_5098_MOESM11_ESM.docx]

**Table ST4.** Summary of gene ontology (GO) term enrichment of skin gene co-expression modules defined by WGCNA, including total number of genes per module, most significant GO term, and major biological process clusters determined using ReViGO. Significant correlations with infection status, density treatment, time point, and fish standard length indicated (+; positive correlation, -; negative correlation).

| **Module** | **No. genes** | **Infection** | **Density** | **Time** | **Length** | **Top GO** | **ReViGO groups** | **Infection & stress related terms** |
| --- | --- | --- | --- | --- | --- | --- | --- | --- |
| SK1 | 5105 | **-** | **+** | **+** | **+** | Immune response | Immune response, retrograde axon cargo transport, lipid metabolism, regulation of GTPase activity | Antigen processing and presentation of peptide antigen via MHC class I, T cell mediated immunity, regulation of stress-activated protein kinase signalling cascade, leukocyte migration |
| SK2 | 1031 | **-** |  | **+** |  | Regulation of synaptic plasticity | Regulation of synaptic plasticity, tyrosine metabolism, cell adhesion, extracellular matrix organization | Circadian regulation of gene expression, photoperiodism |
| SK3 | 3385 | **+** |  |  |  | Intracellular signal transduction | Intracellular signal transduction, protein phosphorylation, endocytosis, actin cytoskeleton organization | I-kappaB kinase/NF-kappaB signalling, antigen processing and presentation of exogenous peptide antigen via MHC class II |
| SK4 | 2411 |  | **-** |  |  | RNA processing | RNA processing, intracellular transport, ribonucleoprotein complex biogenesis, regulation of cell aging | Type I interferon production, cellular response to stress |
| SK5 | 192 | **-** | **-** |  | **+** | Metal ion transport | Metal ion transport, sensory perception of sound, purine ribonucleoside catabolism, membrane tubulation | N/A |
| SK6 | 1561 | **-** | **+** |  |  | Regulation of gene expression | Regulation of nucleic acid templated transcription, tissue homeostasis, chromatin organization, cell migration | Interleukin-12 production, regulation of wound healing, negative regulation of circadian rhythm |
| SK7 | 289 | **-** |  |  |  | Oxidation-reduction process | Oxidative phosphorylation, proton transport, carbohydrate derivative metabolism, cellular metabolism | N/A |
| SK8 | 763 | **-** |  |  | **+** | Energy derivation by oxidation of organic compounds | Energy derivation by oxidation of organic compounds, monovalent inorganic cation transport, Toll signalling pathway, coenzyme biosynthesis | Toll signalling pathway |
| SK9 | 1018 | **-** |  |  |  | Cell surface receptor signalling pathway | Cell surface receptor signalling pathway, taurine biosynthesis, cell-cell adhesion, zymogen activation | N/A |
| SK10 | 1311 | **+** |  |  | **+** | Autophagy | Regulation of responses to reactive oxygen species, autophagy, clathrin-mediated endocytosis, vacuole organization | Mast cell migration |
| SK11 | 157 | **+** |  |  | **-** | Muscle attachment | Muscle attachment, response to toxic substance, mast cell activation, gas transport | Mast cell activation, myeloid leukocyte activation |
| SK12 | 553 | **-** |  |  |  | Cell cycle | Cell cycle, chromosome organization, regulation of chromosome segregation, vesicle transport along microtubule | N/A |
| SK13 | 192 |  | **-** |  |  | RNA processing | RNA processing, positive regulation of microtubule polymerization or depolymerization, cellular localization, cellular metabolism | Cortisol secretion |
| SK14 | 1774 | **+** |  | **-** |  | Single-organism metabolic process | Cellular lipid metabolism, proteolysis, negative regulation of hydrolase activity, proteasome assembly | T cell costimulation, immune response-regulating cell surface receptor signalling pathway involved in phagocytosis, mast cell mediated immunity, response to yeast, myeloid leukocyte mediated immunity, circadian rhythm |
