# Supplementary material for: Transcriptomic response to parasite infection in Nile tilapia (Oreochromis niloticus) depends on rearing density
Source: BMC Genomics. 2018 Oct 1;19:723. doi: 10.1186/s12864-018-5098-7 (PMC6167859; doi:10.1186/s12864-018-5098-7)
Supplement: Supplementary file 12 — Principal Component Analysis plots of gene expression: Principal components analysis (PCA) plots of total gene expression profiles in A) gill, and B) skin of uninfected (CTL) and Saprolegnia-challenged (INF) Oreochormis niloticus at two stocking densities (H; high, L; low) and time points (24; 24 h post-exposure, 48; 48 h). (DOCX 16 kb) [file 12864_2018_5098_MOESM12_ESM.docx]

**Table ST5.** Summary of gene ontology (GO) term enrichment of gill gene co-expression modules defined by WGCNA, including total number of genes per module, most significant GO term, and major biological process clusters determined using ReViGO. Significant correlations with infection status, density treatment, time point, and fish standard length indicated (+; positive correlation, -; negative correlation).

| **Module** | **No. genes** | **Infection** | **Density** | **Time** | **Length** | **Top BP GO term** | **ReViGO groups** | **Infection & stress related terms** |
| --- | --- | --- | --- | --- | --- | --- | --- | --- |
| G1 | 3911 | **-** |  |  |  | Cell communication | Cell communication, lymph vessel development, peptidyl-tyrosine modification, Golgi vesicle budding | Immune response, antigen processing and presentation, chemokine-mediated signalling pathway, response to steroid hormone |
| G2 | 2233 | **+** |  |  |  | Gene expression | Regulation of cellular protein metabolism, cellular component biogenesis, heterocycle metabolism, instracellular transport | Epidermis morphogenesis, response to stress, inflammatory response, mast cell migration, negative regulation of circadian rhythm, T cell proliferation, leukocyte migration, wound healing |
| G3 | 378 |  | **+** |  |  | N/A | N/A | N/A |
| G4 | 1653 | **-** |  |  |  | Regulation of signalling | Regulation of response to stimulus, single-multicellular organism process, single-organism developmental process, cell migration | Stress-activated protein kinase signalling cascade, interleukin-12 production |
| G5 | 748 | **-** |  |  |  | Negative regulation of TOR signalling | Embryonic hemopoiesis, negative regulation of TOR signalling, iron ion transport, peptidyl-serine phosphorylation | Interleukin-12 production |
| G6 | 1139 | **+** |  |  |  | Negative regulation of actin filament polymerization | Negative regulation of actin filament polymerization, nitrogen compound transport, mannosylation, muscle cell fate specification | N/A |
| G7 | 1608 | **-** |  | **+** |  | Regulation of biological process | Regulation of nucleic acid-templated transcription, striated muscle cell differentiation, spindle midzone assembly, inositol metabolism | Positive regulation of stress-activated protein kinase signalling cascade |
| G8 | 233 |  | **-** | **-** |  | Negative regulation of hyaluronan biosynthetic process | Negative regulation of carbohydrate metabolism, intracellular transport, N-terminal protein amino acid acetylation, hyaluronan biosynthesis | Antigen processing and presentation of exogenous peptide antigen via MHC class II, regulation of defense response to virus, immune system development, T cell co-stimulation |
| G9 | 127 | **-** | **-** | **+** |  | Circadian regulation of gene expression | Circadian regulation of gene expression, photoperiodism, heme transmembrane transport, rhythmic process | N/A |
| G10 | 519 | **+** |  | **+** |  | ATP metabolic process | Oxidative phosphorylation, proton transport, regulation of NIK/NF-kappaB signalling, post-chaperonin tubulin folding pathway | Response to stress |
| G11 | 4623 | **+** |  | **-** |  | NcRNA metabolic process | Monosaccharide metabolism, ncRNA metabolism, intracellular transport, ribosome biogenesis | Response to fungus, leukocyte mediated immunity, regulation of T cell mediated immunity, mast cell activation, type I interferon production, macrophage differentiation |
