# Supplementary material for: Transcriptomic response to parasite infection in Nile tilapia (Oreochromis niloticus) depends on rearing density
Source: BMC Genomics. 2018 Oct 1;19:723. doi: 10.1186/s12864-018-5098-7 (PMC6167859; doi:10.1186/s12864-018-5098-7)
Supplement: Supplementary file 12 [file 12864_2018_5098_MOESM5_ESM.docx]

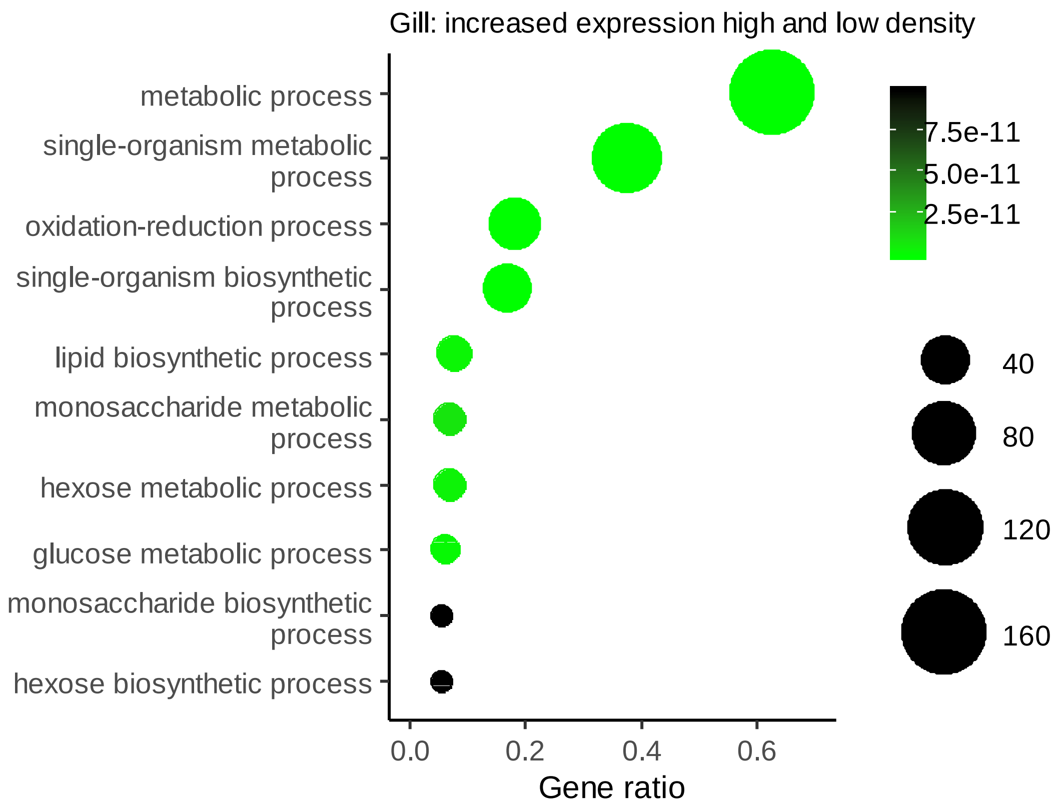
Gill: increased expression high and low density
metabolic process
single-organism metabolic process
oxidation-reduction process
single-organism biosynthetic process
lipid biosynthetic process
monosaccharide metabolic process
hexose metabolic process
glucose metabolic process
monosaccharide biosynthetic process
hexose biosynthetic process
0.0
0.2
0.4
0.6
Gene ratio
7.5e-11
5.0e-11
2.5e-11
40
80
120
160

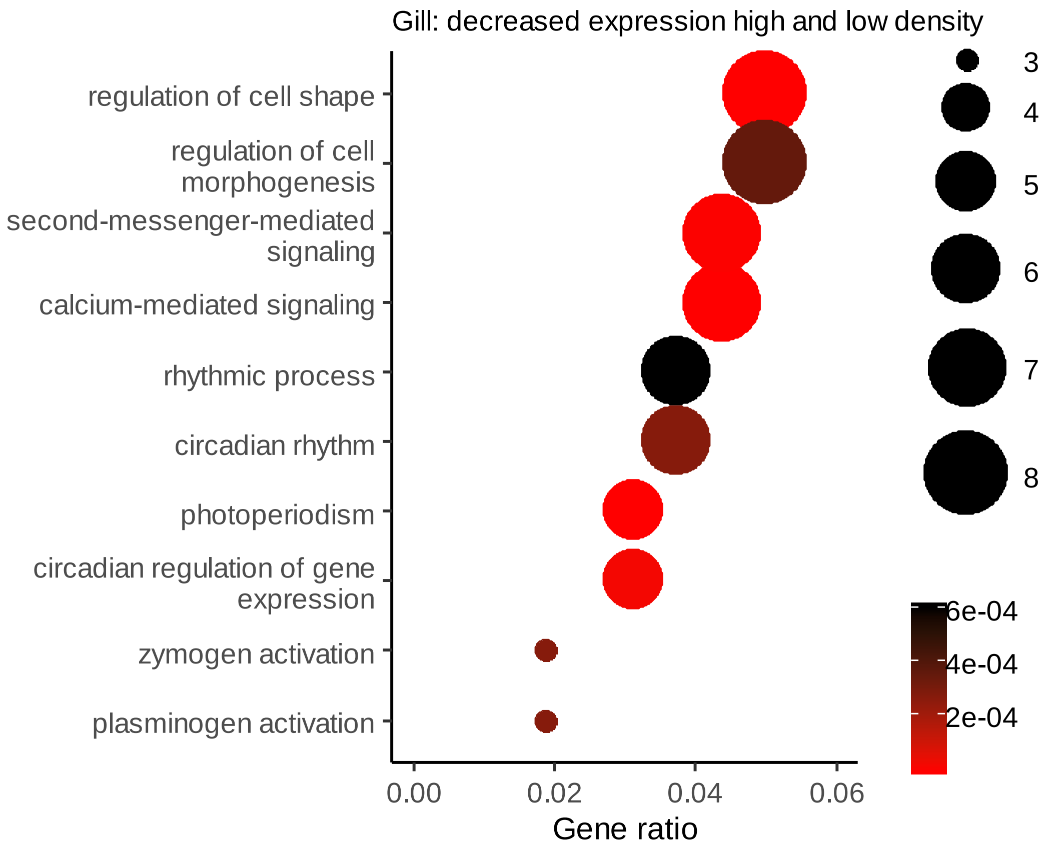
Gill: decreased expression high and low density
regulation of cell shape
regulation of cell morphogenesis
second-messenger-mediated signaling
calcium-mediated signaling
rhythmic process
circadian rhythm
photoperiodism
circadian regulation of gene expression
zymogen activation
plasminogen activation
0.00
0.02
0.04
0.06
Gene ratio
3
4
5
6
7
8
6e-04
4e-04
2e-04

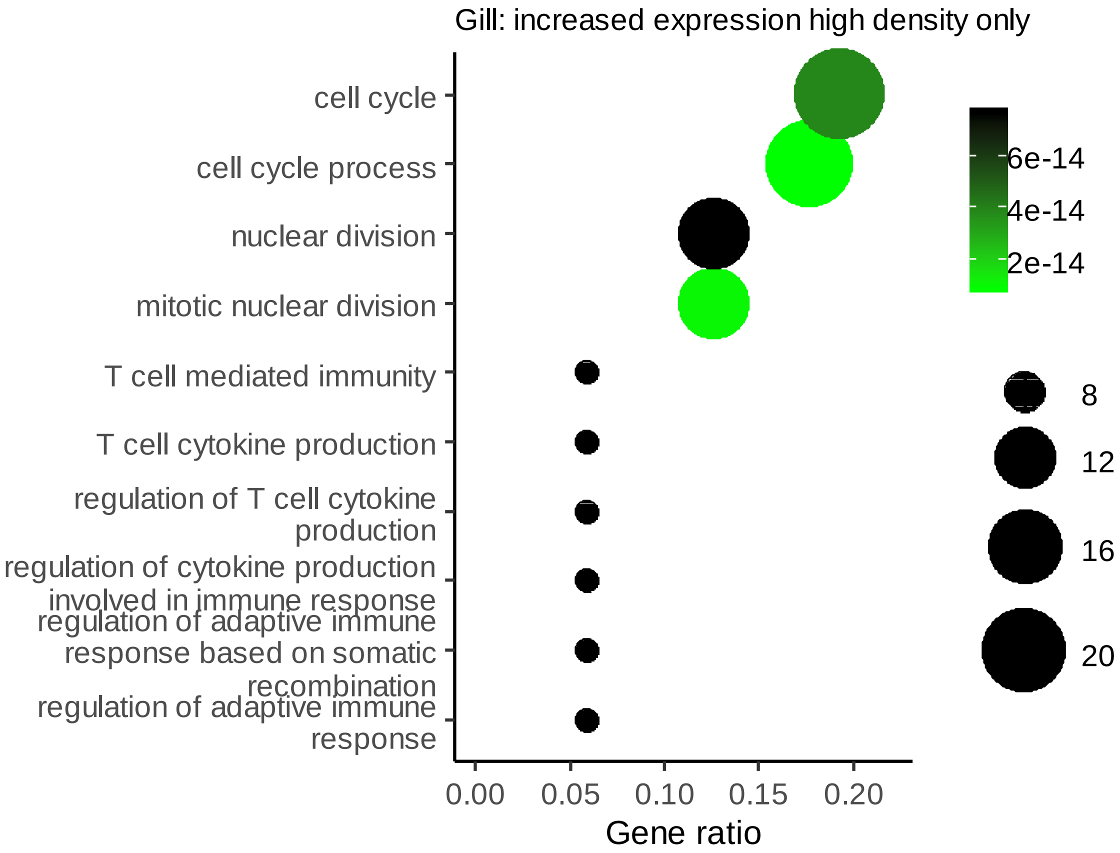
Gill: increased expression high density only
cell cycle
cell cycle process
nuclear division
mitotic nuclear division
T cell mediated immunity
T cell cytokine production
regulation of T cell cytokine production
regulation of cytokine production involved in immune response
regulation of adaptive immune response based on somatic recombination
regulation of adaptive immune response
0.00
0.05
0.10
0.15
0.20
Gene ratio
6e-14
4e-14
2e-14
8
12
16
20

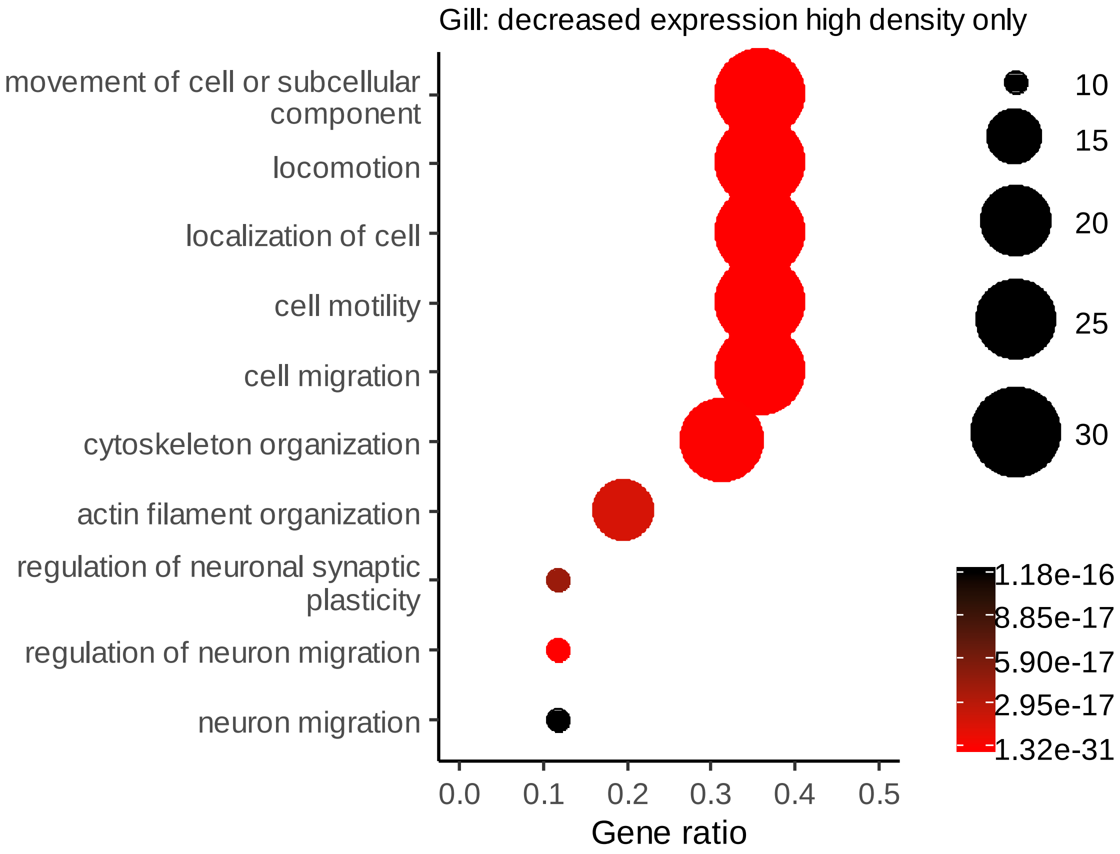
Gill: decreased expression high density only
movement of cell or subcellular component
locomotion
localization of cell
cell motility
cell migration
cytoskeleton organization
actin filament organization
regulation of neuronal synaptic plasticity
regulation of neuron migration
neuron migration
0.0
0.1
0.2
0.3
0.4
0.5
Gene ratio
10
15
20
25
30
1.18e-16
8.85e-17
5.90e-17
2.95e-17
1.32e-31

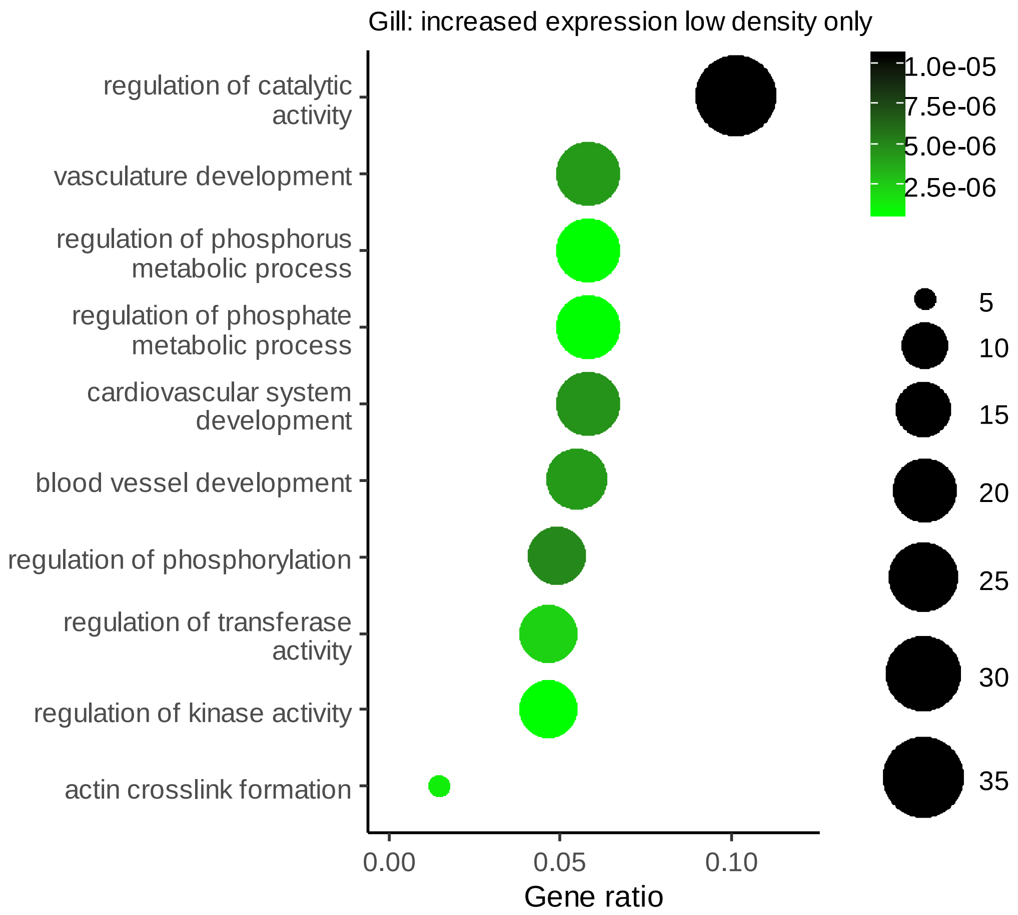
Gill: increased expression low density only
regulation of catalytic activity
vasculature development
regulation of phosphorus metabolic process
regulation of phosphate metabolic process
cardiovascular system development
blood vessel development
regulation of phosphorylation
regulation of transferase activity
regulation of kinase activity
actin crosslink formation
0.00
0.05
0.10
Gene ratio
1.0e-05
7.5e-06
5.0e-06
2.5e-06
5
10
15
20
25
30
35

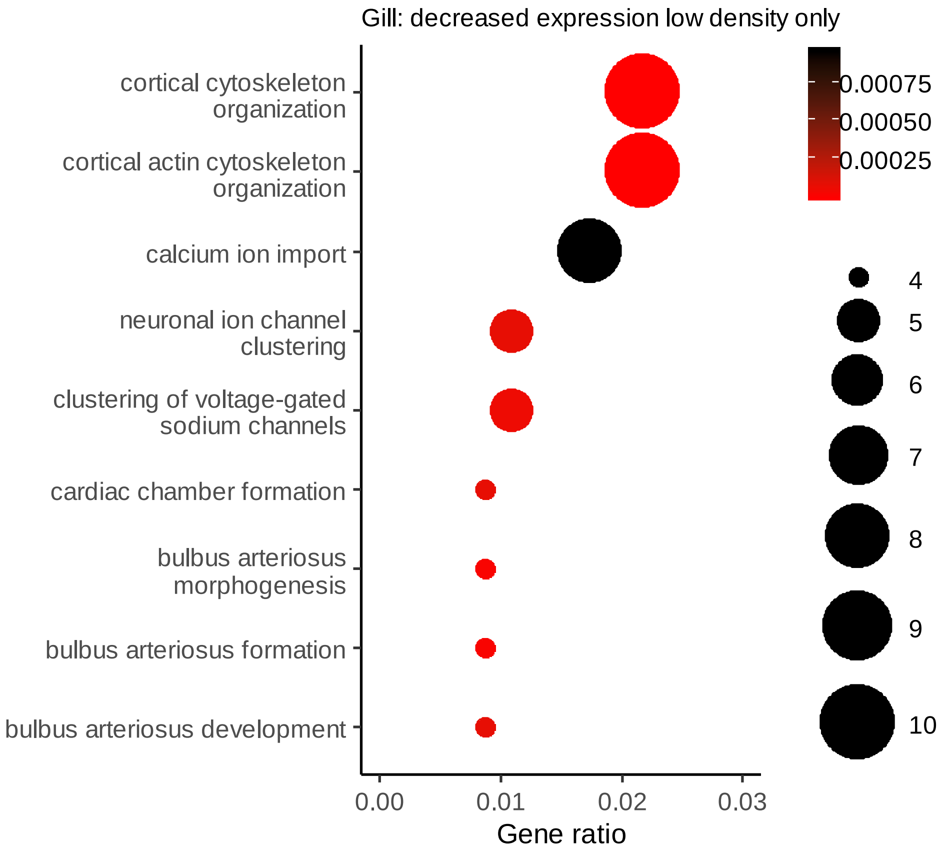
Gill: decreased expression low density only
cortical cytoskeleton organization
cortical actin cytoskeleton organization
calcium ion import
neuronal ion channel clustering
clustering of voltage-gated sodium channels
cardiac chamber formation
bulbus arteriosus morphogenesis
bulbus arteriosus formation
bulbus arteriosus development
0.00
0.01
0.02
0.03
Gene ratio
0.00075
0.00050
0.00025
4
5
6
7
8
9
10
